# Effects of Intermittent and Continuous Static Stretching on Range of Motion and Musculotendinous Viscoelastic Properties Based on a Duration-Matched Protocol

**DOI:** 10.3390/ijerph182010632

**Published:** 2021-10-11

**Authors:** Kensuke Oba, Mina Samukawa, Yosuke Abe, Yukino Suzuki, Miho Komatsuzaki, Satoshi Kasahara, Tomoya Ishida, Harukazu Tohyama

**Affiliations:** 1 Graduate School of Health Sciences, Hokkaido University, Sapporo 060-0812, Japan; obaknsk@gmail.com (K.O.); ab91mtsog639@gmail.com (Y.A.); atty1616@gmail.com (M.K.); 2 Department of Rehabilitation, Hitsujigaoka Hospital, Sapporo 004-0021, Japan; 3 Faculty of Health Sciences, Hokkaido University, Sapporo 060-0812, Japan; kasahara@hs.hokudai.ac.jp (S.K.); t.ishida@hs.hokudai.ac.jp (T.I.); tohyama@med.hokudai.ac.jp (H.T.); 4 Department of Rehabilitation, Hokushin Orthopaedic Hospital, Sapporo 060-0908, Japan; yukino.suzuki0825@gmail.com

**Keywords:** ankle, joint flexibility, stiffness, constant torque stretching, passive resistive torque

## Abstract

The different effects of intermittent and continuous stretching on the mechanical properties of the musculotendinous complex have been unclear. This study aimed to compare the effects of intermittent and continuous stretching for the same duration on the range of motion (ROM), passive resistive torque (PRT), and musculotendinous stiffness (MTS) of ankle plantar flexors. Eighteen healthy young men participated in the study. Intermittent (four sets × 30 s) and continuous stretching (one set × 120 s) were performed in random orders on two separate days. Both stretching protocols were conducted using a dynamometer with a constant torque applied. ROM and PRT were determined using a dynamometer, and MTS was calculated using the torque–angle relationship measured before and after stretching. Two-way repeated measures analysis of variance was performed for all parameters. Both intermittent and continuous stretching significantly increased ROM and decreased PRT and MTS (*p* < 0.05). Intermittent stretching led to greater changes in ROM and PRT than continuous stretching. However, the reduction in MTS did not differ between the two conditions. These results suggest that intermittent stretching is more effective in increasing ROM and changing the mechanical properties of the musculotendinous complex.

## 1. Introduction

Static stretching (SS) increases range of motion (ROM) and reduces musculotendinous stiffness (MTS) [1]. SS has been used in various situations, such as sports, rehabilitation, and health promotion. SS involves holding a muscle group at an end range of motion (ROM) for a certain time. In a clinical setting, the intermittent stretching protocol (INT) was commonly performed with short-time repetitive stretching. The continuous stretching protocol (CON) is a steady stretching protocol. The total stretch duration is considered an important factor affecting the ROM increase and MTS reduction [2,3]. Until now, any comparison of INT and CON was reported with the maximum ROM change as an outcome [4,5,6,7], and the results were not consistent, even though the total duration was the same. The ROM measurement comprises sensory effects, such as the pain threshold, stretch tolerance, and reflex activation of agonist muscles [8], as well as mechanical effects [9]. Furthermore, other factors, such as age, gender, sports level, stretch duration, and target muscles and/or joints, are also considered to affect the results. Passive resistive torque (PRT) and MTS represent the mechanical properties of musculotendinous complexes and eliminate stretch-induced sensory effects. Therefore, it is crucial that both mechanical properties of the musculotendinous complex and ROM be determined. Recent studies of INT [10] or CON [11] showed that neither two sets of 30 s stretching nor one set of 60 s stretching changed MTS. The mechanical property changes in the musculotendinous complex between INT and CON remain unclear. To the best of our knowledge, no previous studies have directly compared the stretching effects of INT and CON on the mechanical properties of the muscle tendon complex.

Therefore, the current study aimed to determine the effects of INT and CON with a matched duration on the musculotendinous extensibility. From the results of our previous study [1], constant torque stretching was effective for musculotendinous extensibility with a strong intensity. The next question arising is that of the stretch duration with/without a rest interval. Repetitive passive strain is considered to cause a rapid redistribution of polysaccharides and water within the collagen framework, changing the muscle thixotropy and viscosity [12]. Due to the theory by McNair et al. [12], we hypothesized that INT would be more effective in increasing ROM and reducing PRT and MTS than CON, even though the total stretch duration was equally applied.

## 2. Materials and Methods

### 2.1. Experimental Protocol

The current study had a cross-over design, and two stretching protocols (INT and CON) were compared to ascertain the effects on musculotendinous extensibility. Participants were instructed to visit the laboratory room three times. The first visit was to familiarize them with the study protocols, and to measure the maximum tolerable torque threshold for stretch intensity. For the second and the third visits, either INT or CON was conducted in a random order at the same time of the day (± 2 h) with an interval of at least 48 h. Before and after stretching, the maximum ankle dorsiflexion ROM, PRT, and MTS of the ankle plantar flexors were measured.

### 2.2. Participants

Eighteen young healthy male participants aged 22.7 ± 1.4 years (height: 175.4 ± 4.4 cm and body mass: 66.4 ± 8.5 kg) volunteered to participate in this study. The ankle plantar flexors of the participants’ dominant leg were stretched. Participants with no current lower limb injuries and no history of neuromuscular diseases were included. Participants who regularly performed flexibility exercises or resistance training were excluded. The participants were instructed to refrain from participating in any intensive exercise for 24 h prior to the commencement of the experiment. This study was approved by the local institutional review board (approval number: 19-66). The participants were provided with the research details, and written informed consent was obtained from each participant prior to the study. All procedures were performed in accordance with the principles of the Declaration of Helsinki and the present study protocol was approved by the Institutional Review Board of Faculty of Health Sciences, Hokkaido University.

### 2.3. ROM and PRT Measurements

Ankle joint angles and torque values were determined using a Biodex System 3 isokinetic dynamometer (Biodex Medical Systems, Inc., Shirley, NY, USA). The participant was placed in a supine position with the right knee in full extension, and the lateral malleolus of the fibula was aligned with the axis of the dynamometer. The foot was firmly fixed onto a footplate with a Velcro strap to prevent movement during passive ankle dorsiflexion. The neutral angle of the ankle joint was set perpendicular to the footplate. The positive and negative values indicate ankle dorsiflexion and plantar flexion, respectively. During the maximum tolerable torque threshold measurement during familiarization, the ankle was passively dorsiflexed from 20° of plantar flexion. The participants pressed the safety trigger button when they attained the maximum stretch feeling without pain. The angular velocity in the lever arm was moved at 1°/s to obtain the maximum stretch feeling and avoid stretch reflex or muscle activity [13,14]. The same settings were used for both INT and CON. Before and after the stretching intervention, the ankle joint angle and torque were measured with passive dorsiflexion from 20° of plantar flexion to the angle when the maximum tolerable torque threshold was met. The practice sessions were performed twice before the measurement. The participants were instructed to relax the lower leg during the measurement.

The ankle joint angles and torque signals were sampled simultaneously at 1000 Hz using MyoSystem 1200 (Noraxon USA, Inc., Scottsdale, AZ, USA). All signals were processed offline using the MATLAB software (MathWorks, Natick, MA, USA). The ankle joint angles and torque signals were low-pass filtered at 10 Hz, and a gravity correction of the limb was performed using the torque data at the starting position [15]. PRT represents the amount of passive resistance produced by the musculotendinous complex at the joint angle. ROM was measured at a predetermined maximum tolerable torque threshold [15]. MTS was calculated as the slope of the relationship between torque and joint angle. PRT and MTS were quantified using a fourth-order polynomial regression model that was fitted to passive torque–angle curves [16]. MTS and PRT were calculated at the same ankle dorsiflexion angle before and after the stretching. The calculated ankle dorsiflexion angle was defined as the maximum ankle dorsiflexion angle before stretching.

### 2.4. Duration-Matched Stretch Protocol

SS was performed using a Biodex isokinetic dynamometer. The maximum tolerable torque threshold was used to determine the intensity of the constant torque stretching. The ankle joint was passively dorsiflexed up to the maximum tolerable torque threshold and was controlled to maintain a constant torque load with more than 95% of the maximum tolerable torque threshold for stretching. Based on duration-matched protocols, the INT protocol consisted of 4 sets of 30 s stretching with a 15 s interval between each set. The CON protocol consisted of 120 s of continuous stretching. Architectural changes were found after 120 s of stretching [8], and other studies with 120 s stretching protocols have shown changes in the mechanical properties of the musculotendinous complex [17,18]. From these study results, a total stretch duration of 120 s was conducted in this research.

### 2.5. Statistical Analysis

The sample size required for a two-way repeated measures analysis of variance (ANOVA; effect size [ES] = 0.40 [large], α error = 0.05, and power = 0.80) was calculated using the G*Power 3.1 software (Heinrich Heine University Düsseldorf, Düsseldorf, Germany), and the minimum number of participants was 14 for this study. All statistical analyses were performed using the Statistical Package for the Social Sciences software (version 21.0; IBM Japan Co., Tokyo, Japan). A Shapiro–Wilk test was used to assess the normal distribution of all data. Two-way repeated measures ANOVA [condition (INT and CON) × time (before and after)] was performed to determine any significant differences in all outcomes. When a significant effect was identified, paired t-tests with Bonferroni corrections were performed to compare all parameters. The percent change in each parameter before and after stretching was calculated. ES was calculated using partial eta-squared values (η^2^_p_) for repeated measures. Cohen’s d was calculated to determine the ES for the magnitudes of the differences. ES was defined as small (d = 0.2), moderate (d = 0.5), or large (d = 0.8) [19]. In terms of the test–retest reliability, the intraclass correlation coefficient (ICC_1,1_) and the standard error of measurement (SEM) were calculated for each condition as follows: ROM (ICC_1,1_ = 0.94; 95% confidence interval [CI] = 0.86 – 0.98; SEM = 1.6), MTS (ICC_1,1_ = 0.93; 95% CI = 0.84 – 0.98; SEM = 0.15), and PRT (ICC_1,1_ = 0.99; 95% CI = 0.99 – 1.00; SEM = 0.42). Statistical significance was set at *p* < 0.05, and all data were presented as mean ± standard deviation.

## 3. Results

### 3.1. ROM

The two-way interaction (condition × time) was significant (*p* < 0.01; η^2^_p_ = 0.39) (Table 1). ROM was significantly increased after INT (ES = 0.42; *p* < 0.01) and CON (ES = 0.36; *p* < 0.01). Before stretching, there were no significant differences in ROM (*p* = 0.15). Nevertheless, ROM significantly differed between INT and CON after stretching (*p* = 0.04).

### 3.2. PRT

The two-way interaction (condition × time) was significant (*p* < 0.01; η^2^_p_ = 0.56) (Table 1). PRT was significantly decreased after INT (ES = 0.37; *p* < 0.01) and CON (ES = 0.30; *p* < 0.01). Before stretching, there were no significant differences in PRT (*p* = 0.48). Nevertheless, the PRT significantly differed between INT and CON after stretching (*p* < 0.01).

### 3.3. Musculotendinous Stiffness

The two-way interaction (condition × time) (*p* = 0.99; η^2^_p_ = 0.00) and the main effect of condition were not significant (*p* = 0.31; η^2^_p_ = 0.06) (Table 1). Nevertheless, a main effect of time was observed (*p* < 0.01; η^2^_p_ = 0.67). MTS significantly decreased after INT (ES = 0.26; *p* < 0.01) and CON (ES = 0.27; *p* < 0.01). However, there were no significant differences in terms of MTS between the conditions before and after stretching (*p* = 0.41 and 0.25, respectively).

## 4. Discussion

The present study investigated differences in the effects of INT and CON on the ROM, PRT, and MTS of ankle plantar flexors based on a duration-matched protocol. Our study showed that a similar reduction in MTS was observed with the same stretch duration (120 s). Another finding was that INT induced a significant increase in ROM and decrease in PRT compared with CON. These results suggested that INT was more effective than CON with the same stretch duration for changes in ROM and mechanical properties, such as PRT.

This is the first study to compare the effects of INT and CON on both ROM and the mechanical properties with INT and CON. The main finding of this study was that INT resulted in a greater change in ROM and PRT than CON. These results are consistent with those of a previous study [6]. The ankle dorsiflexion ROM was measured at a predetermined maximum tolerable torque threshold. Thus, ROM was determined at the same passive torque threshold before and after stretching. Therefore, INT led to a larger ROM increase than CON because the decrease in PRT was greater in INT than in CON. Several other studies have reported different results that led to a similar increase in ROM between INT and CON [4,5]. In these studies, ROM was measured by subjective stretch feeling, so stretch-induced sensory changes might have been involved. Considering our results that INT induced larger changes in the mechanical properties of the musculotendinous complex, it is possible that CON is influenced more by sensory factors than INT because the ROM improvement was similar between INT and CON in the previous research results [4,5]. Trajano et al. [7] showed that only CON significantly increased ROM, which was different from our results. These conflicting results could be due to the stretch intensity and torque adjustment. In the previous study carried out by Trajano et al. [7], the ankle joint was passively dorsiflexed until the passive torque had attained 90% of the maximal tolerable torque threshold. Then, the ankle joint angle was adjusted to maintain the level of passive torque to within 5 Nm of the 90% threshold. In our study, the ankle joint was passively dorsiflexed until the passive torque reached 100% of the maximal tolerable torque threshold. It was then maintained at 95% or more. Therefore, the stretch intensity of our study was stronger than that of the Trajano study [7]. Furthermore, the torque adjustment was controlled according to the participant and the load was higher in our study than the Trajano study [7], which may also have contributed to the conflicting results.

PRT indicates the torque of the passive resistance produced by not only the musculotendinous complex, but also by the joint capsule, ligaments, and connective tissues around the joint [20]. In this study, ROM was determined using the ankle dorsiflexion angle with the maximum tolerable torque threshold before the stretching protocols. That is to say, ROM tests were performed with the same torque values applied before and after stretching. Our study results found that INT led to larger PRT decreases and greater ROM increases. The cyclic strain with a repetitive passive motion is considered to cause a rapid redistribution of polysaccharides and water within the collagen framework, leading to changes in muscle thixotropy and viscosity [12]. Therefore, INT is thought to be more effective for changing mechanical properties than CON. As for the rest interval between the stretches [21,22], no rest (0 s) intervals induced greater changes in muscle stiffness than 30 s intervals [21], whereas similar changes were shown among three different rest intervals (10 s, 30 s, and 90 s) [22]. Therefore, further consideration of the effects of rest intervals with INT is needed.

The MTS reduction was not significantly different between INT and CON conditions with the same total duration. MTS reflects the slope of the relationship between the passive torque and joint angle. Therefore, PRT and MTS exhibit different mechanical characteristics [23]. Our present findings suggest that the change in MTS was dependent on the total stretch duration. However, we could not determine which tissues and/or structures responded to changes in PRT and MTS in this study. A direct observation using ultrasound may confirm this.

The current study had a few methodological limitations that should be addressed. Only healthy young men were enrolled in the present study. We did not specify information on their activity levels and exercise habits. Population characteristics, such as sex, age, activity level, and exercise habits, may influence the stretching effects.

The present study revealed that INT was more effective in increasing ROM and mechanical properties than CON. For athletic performance, CON decreased the jump height and induced muscle fatigue compared with INT [4,24,25]. These studies suggest that CON has detrimental effects on muscle power performance. Therefore, INT is recommended for an increase in ROM and is also suitable for preparation exercises in clinical and sports settings.

## 5. Conclusions

The present study compared stretching effects with INT (30 s × 4 sets) and CON (120 s × 1 sets) on musculotendinous extensibility. The results showed that INT and CON decreased MTS in a similar manner. INT elicited a larger increase in ROM and decrease in PRT compared with CON. Therefore, INT may be a more effective technique, not only for increasing ROM, but also for changing mechanical properties.

## Figures and Tables

**Table 1:** Changes in ankle dorsiflexion range of motion, passive resistive torque, and musculotendinous stiffness before and after stretching.

	Condition	Before	After	% Change	Effect Size	Interaction
ROM (°)	INT	35.0 ± 7.0	38.1 ± 7.8 ^a,b^	8.6	0.42	*p* < 0.01
	CON	34.2 ± 6.8	36.7 ± 7.3 ^a,b^	7.2	0.36	
PRT (Nm)	INT	35.7 ± 13.5	31.1 ± 11.9 ^a,b^	−12.7	0.37	*p* < 0.01
	CON	35.6 ± 13.5	31.8 ± 12.3 ^a,b^	−10.7	0.30	
MTS (Nm/°)	INT	1.7 ± 0.6	1.5 ± 0.6 ^a^	−8.8	0.26	*p* = 0.99
	CON	1.7 ± 0.6	1.6 ± 0.6 ^a^	−8.4	0.27	

Data are presented as mean ± SD. ^a^ Significant difference between before and after stretching. ^b^ Significant difference between INT and CON.

## Data Availability

The datasets in this study are available on reasonable request to the corresponding author’s email.
